# How Artificial Intelligence Can Help Us Understand Human Creativity

**DOI:** 10.3389/fpsyg.2019.01401

**Published:** 2019-06-19

**Authors:** Fernand Gobet, Giovanni Sala

**Affiliations:** ^1^Department of Psychological Sciences, University of Liverpool, Liverpool, United Kingdom; ^2^Graduate School of Human Sciences, Osaka University, Suita, Japan

**Keywords:** artificial intelligence, bounded rationality, creativity, evolutionary computation, intelligence, simulation, scientific discovery, theory

## Abstract

Recent years have been marked by important developments in artificial intelligence (AI). These developments have highlighted serious limitations in human rationality and shown that computers can be highly creative. There are also important positive outcomes for psychologists studying creativity. It is now possible to design entirely new classes of experiments that are more promising than the simple tasks typically used for studying creativity in psychology. In addition, given the current and future AI algorithms for developing new data structures and programs, novel theories of creativity are on the horizon. Thus, AI opens up entire new avenues for studying human creativity in psychology.

In psychology, research into creativity^1^ has tended to follow well-trodden paths: simple tests of creativity (e.g., alternative uses test), correlations with measures of intelligence, and more recently neural correlates of creativity such as EEG and fMRI (e.g., Weisberg, 2006; Runco, 2014)^2^. One line of research that has been little explored is to use progress in artificial intelligence (AI) to generate tools for studying human creativity.

Developments of AI have been impressive. DeepMind’s AlphaGo has easily beaten the best human grandmasters in Go, a game that for many years had seemed beyond the reach of AI (Silver et al., 2016). IBM’s Watson mastered natural language and knowledge to the point that it outclassed the best human players in Jeopardy! – a game show where contestants have to find the question to an answer (Ferrucci, 2012). Not less impressive, we are now on the brink of having self-driving cars and automated assistants able to book appointment by phone (Smith and Anderson, 2014). These developments raise profound issues about human identity; they also pose difficult but exciting questions about the very nature of human creativity and indeed rationality. But they also present novel opportunities for studying human creativity. Entirely new classes of experiments can be devised, going way beyond the simple tasks typically used so far for studying creativity, and new theories of creativity can be developed.

## Artificial Intelligence Research and Creativity

Using AI for understanding creativity has a long history and is currently an active domain of research with annual international conferences (for reviews, see Meheus and Nickles, 2009; Colton and Wiggins, 2012). As early as 1957, Newell, Simon, and Shaw had programmed Logic Theorist to prove theorems in symbolic logic. Not only did this research lead to an influential theory of problem-solving (Newell et al., 1958) but it also shed important light on human creativity, as Logic Theorist was able to prove some theorems in a more elegant way than Russell and Whitehead, two of the leading mathematicians of the twentieth century (Gobet and Lane, 2015). There are numerous examples of AI creativity in science today (Sozou et al., 2017). For example, at Aberystwyth University, a “robot scientist” specialized in functional genomics not only produced hypotheses independently but also designed experiments for testing these hypotheses, physically performed them and then interpreted the results (King et al., 2004).

In the arts, British abstract painter Harold Cohen all but abandoned a successful career as an artist to understand his own creative processes. To do so, he wrote a computer program, AARON, able to make drawings and later color paintings autonomously (McCorduck, 1990). More recently, several programs have displayed high levels of creativity in the arts. For example, a deep-learning algorithm produced a Rembrandt-like portrait (Flores and Korsten, 2016) and the program Aiva, also using deep learning, composes classical music (Aiva Technologies, 2018). An album of Aiva’s music has already been released, and its pieces are used in films and by advertising agencies. In chess, the program CHESTHETICA automatically composes chess problems and puzzles that are considered by humans as esthetically pleasing (Iqbal et al., 2016).

However, AI has had only little impact on creativity research in psychology (for an exception, see Olteţeanu and Falomir’s, 2015, 2016 work on modelling the Remote Associate Test and the Alternative Uses Test). There is only passing mention if at all in textbooks and handbooks of creativity (e.g., Kaufman and Sternberg, 2006; Runco, 2014), and mainstream research simply ignores it. In our view, this omission is a serious mistake.

## The Specter of Bounded Rationality

AI has uncovered clear limits in human creativity, as is well illustrated by Go and chess, two board games requiring creativity when played competitively. After losing 3–0 against computer program AlphaGo Master in 2017, Chinese Go grandmaster Ke Jie, the world No. 1, declared: “After humanity spent thousands of years improving our tactics, computers tell us that humans are completely wrong… I would go as far as to say not a single human has touched the edge of the truth of Go” (Kahn, 2017). Astonishingly, this version of AlphaGo, which won not only all its games against Ke Jie but also against other leading Go grandmasters, was beaten 89–11 a few months later by AlphaGo Zero, a new version of the program that learns from scratch by playing against itself, thus creating all its knowledge except for the rules of the game (Silver et al., 2016, 2017).

Ke Jie’s remark is echoed by chess grandmasters’ comments (Gobet, 2018). In the second game of his 1997 match against Deep Blue, Kasparov and other grandmasters were astonished by the computer’s sophisticated and creative way of first building a positional advantage and then denying any counter-play for Kasparov. Kasparov’s surprise was such that he accused IBM and the programming team behind Deep Blue of cheating, a charge that he maintained for nearly 20 years. More recently, in the sixth game of the 2006 match between Deep Fritz and world champion Vladimir Kramnik, the computer played a curious rook maneuver that commentators ridiculed as typical of a duffer. As the game unfolded, it became clear that this maneuver was a very creative way of provoking weaknesses on Kramnik’s kingside, which allowed Deep Blue to unleash a fatal offensive on the other side of the board.

In general, these limits in rationality and creativity are in line with Simon’s theory of bounded rationality (Simon, 1956, 1997; Gobet and Lane, 2012; Gobet, 2016a), which proposed that limitations in knowledge and computational capacity drastically constrain a decision maker’s ability to make rational choices. These limits are also fully predictable from what we know from research in cognitive psychology. For example, Bilalić et al. (2008) showed that even experts can be blinded by their knowledge, with the consequence that they prefer standard answers to novel and creative answers, even when the latter are objectively better. Thus, when a common solution comes first to mind, it is very hard to find another one (a phenomenon known as the Einstellung effect). In Bilalić et al.’s chess experiment, the effect was powerful: compared to a control group, the strength of the Einstellung group decreased by about one standard deviation.

The power of long-term memory schemas and preconceptions is a common theme in the history of science and art and has often thwarted creativity. For example, in the early 1980s, the unquestioned wisdom was that stomach ulcers were caused by excess acid, spicy food, and stress. The genius of Marshall and Warren (1984) in their Nobel-winning discovery was to jettison all these assumptions before hypothesizing that a bacterium (helicobacter pylori) was the main culprit. Finding ways to overcome such mind-sets is an important task for fostering human creativity (Gobet et al., 2014), as they are common with normal cognition. In some instances, in order to be creative and explore new conceptual spaces, it is necessary to break these mind-sets, either by inhibiting some specific concepts or groups of concepts, or by eschewing concepts altogether. AI systems can use a large variety of different methods – some similar to those used by humans, some entirely dissimilar. Thus, they are less likely to be subject to such mind-sets and could provide humans with useful alternatives for developing creative products.

## Artificial Intelligence Offers Novel Methods for Studying Creativity

When considering the literature on creativity in psychology, it is hard to escape the feeling that something is amiss in this field of research. A considerable amount of research has studied simple tasks that are remote from real creativity in the arts and science – for example, alternative uses task, word generation task, and insight problems (see e.g., Runco, 2014) – but it is at the least debatable whether these tasks tell us much about real creativity. As support for this critique of the lack of ecological validity of many tasks used in the field, numerous experiments have found that these tasks correlate more with general intelligence (g) and verbal intelligence than with real-world creativity (Wallach, 1970; Silvia, 2015). In addition, in their review of the literature, Zeng et al. (2011) conclude that divergent-thinking tests suffer from six major weaknesses, including poor predictive, ecological, and discriminant validities. (For a more positive evaluation, see Plucker and Makel, 2010.) While some researchers have developed tasks that map more directly into the kind of tasks carried out in real-world creativity – see in particular the research on scientific discovery (Klahr and Dunbar, 1988; Dunbar, 1993) – this approach is relatively underrepresented in research into creativity.

A similar concern can be voiced with respect to experimentation and theory development. Although a fair amount of avenues have been explored – including generation and selection (e.g., Simonton, 1999), heuristic search (e.g., Newell et al., 1962), problem finding (e.g., Getzels and Csikszentmihalyi, 1976), systems theories (e.g., Gruber, 1981), explanations based on intelligence (e.g., Eysenck, 1995), and psychopathological explanations (e.g., Post, 1994) – entire experimental and theoretical spaces have been fully ignored or, in the best case, barely scratched. Clearly, this is due to the limits imposed by human bounded rationality, to which one should add the constraints imposed by the limited time resources available.

AI can help with both empirical and theoretical research. Empirically, it can simulate complex worlds that challenge human creativity; theoretically, it can help develop new theories by inhibiting some concepts (see above), making unexpected connections between known mechanisms or proposing wholly new explanations. Here we focus on scientific discovery, but similar conclusions can be reached for creativity in the arts.

### A New Way of Designing Experiments

AI can be used as a new way to perform experiments on creativity. The central idea is to exploit current technology to design complex environments that can be studied with a creative application of the scientific method. Thus, these experiments go way beyond the simple tasks typically used in creativity research. Rather than studying creativity asking people to generate words that are related to three stimulus words as in the Remote Associates Test (Mednick, 1962), one studies it by asking participants to find the laws of a simulated world. This is of course what Dunbar, Klahr, and others did in earlier experiments (Klahr and Dunbar, 1988; Dunbar, 1993). The key contribution here is to propose to use much more complex environments, including environments where the presence of intelligent agents approximates the complexity of studying phenomena affected by humans, as is the case in psychology and sociology. Thus, where standard programming techniques are sufficient for simulating physical worlds with no intelligent agents, AI techniques make it possible to simulate much more complex worlds, which incorporate not only physical and biological laws, but also psychosocial laws. In both cases, the participants’ task is to reverse-engineer at least some of the laws of the domains – that it to make scientific discoveries about these domains. Thus, for example, participants must devise experiments for understanding the learning mechanisms of agents inhabiting a specific world. The mechanisms and laws underpinning these worlds can be similar to those currently postulated in science, or wholly different with new laws of physics, biology, or psychology. In that case, the situation is akin to scientists exploring life on a new planet.

These environments can be used with several goals in mind. First, they can test current theories of creativity and scientific discovery. The worlds can be designed in such a way that their understanding is facilitated by the mechanisms proposed by some theories as opposed to others (e.g., heuristic search might be successful, but randomly generating concepts might not, or *vice versa*). Additional questions include whether participants adapt their strategy as a function of the results they obtain and whether they develop new experimental designs where necessary. Second, these environments can be used to observe new empirical phenomena related to creativity, such as the generation of as yet unknown strategies. New phenomena are bound to occur, as the complexity of the proposed tasks is larger by several orders of magnitude than the tasks typically studied in psychology.

A third use is to identify creative people in a specific domain, for example in biology or psychology. As creativity is measured in a simulated environment that is close to the target domain, one is more likely to correctly identify individuals that might display creativity in the domain. If one wishes, one can correlate performance in the task and other behavioral measures with standard psychological measures such as IQ, motivation, and psychoticism.

A final use is to train people to be creative in a specific domain. Variables in the environment can be manipulated such that specific skills are taught, for example the efficient use of heuristics or standard research methods in science. The difficulty of finding laws can be manipulated as well: from a clear linear relation between two variables to non-linear relations between several variables with several sources of noise. The reader will have noticed that such environments are not dissimilar from some video games, and this game-like feature can be used to foster enjoyment and motivation, and thus learning.

Please note that we make no claim that training creativity in one domain will provide something like general creativity, as is sometimes proposed in the literature (e.g., De Bono, 1970). There is now very strong experimental evidence that skills acquired in a domain do not generalize to new domains sharing few commonalities with the original one (Gobet, 2016b; Sala and Gobet, 2017a), and this conclusion almost certainly also applies to creativity. One possible reason for this lack of far transfer is that expertise relies on the ability of recognizing patterns that are specific to a domain (Sala and Gobet, 2017b). It is possible to speculate that being creative relies, at least in part, on recognizing rare domain-specific patterns in a problem situation. For example, to go back to the example of discovering that stomach ulcers are caused by bacteria, Warren recognized the presence of bacteria in gastric specimens he studied with a microscope, although this was not expected as it was thought that the stomach was a sterile environment inhospitable for bacteria (Thagard, 1998). However, we do recognize that this is a hypothesis that should be tested, and it could turn out that, in fact, creativity is a general ability. This is an empirical question that can only be settled with new experiments, and the methods proposed in this paper may contribute to its answer.

### Automatic Generation of Theories

As noted above, human bounded rationality has the consequence that humans only explore a very small number of subspaces within the space of all possible theories, and even these subspaces are explored only sparsely. Mind-sets and other biases mean that even bad hypotheses are maintained while more promising ones are ignored. AI can help break these shackles.

The subfield of AI known as computational scientific discovery has been active for decades, spearheaded by Herbert Simon’s seminal work (Newell et al., 1962; Bradshaw et al., 1983). The aim is precisely to develop algorithms that can produce creative behavior in science, either replicating famous scientific discoveries or making original contributions (for a review, see Sozou et al., 2017). Due to space constraints, we limit ourselves to the description of only one approach – Automatic Generation of Theories (AGT) (Lane et al., 2014) – which is particularly relevant to our discussion as it excels in avoiding being stuck in local minima, contrary to human cognition which is notably prone to mind-sets, Einstellung effects, and other cognitive biases. In a nutshell, the central ideas of AGT are (1) to consider theories as computer programs; (2) to use a probabilistic algorithm (genetic programming) to build those programs; (3) to simulate the protocols of the original experiments; (4) to compare the predictions of the theories with empirical data in order to compute the quality (fitness) of the theories; and (5) to use fitness to evolve better theories, using mechanisms of selection, mutation, and crossover. Simulations have shown that the methodology is able to produce interesting theories with simple experiments. With relentless progress in technology, it is likely that this and other approaches in artificial scientific discovery will provide theoretical explanations for more complex human behaviors, including creativity itself.

### Challenges

The two uses of AI proposed in this paper for studying creativity in psychology are not meant to replace current methods, but to add to the arsenal of theoretical concepts and experimental techniques available to researchers. Nor are they proposed as magic bullets that will answer all questions related to creativity. Our point is that these uses of AI present potential benefits that have been overlooked by psychologists studying creativity.

As any new approach, these uses raise conceptual and methodological challenges. Regarding the proposed method for collecting data, challenges include the way participants’ results will be scored and compared, and how they will be used to test theories. A related challenge concerns the kind of theory suitable to account for these data; given the complexity and richness of the data, it is likely that computational models will be necessary – possibly models generated by the second use of AI we proposed.

Similarly, using AI for generating theories raises interesting practical and theoretical questions. Will the generated theories be understandable to humans, or will they only be black boxes providing correct outputs (predictions) given a description of the task at hand and other kind of information such as the age of the participants? Will their structure satisfy canons of parsimony in science? How will they link epistemologically to other theories in psychology, for example theories of memory and decision-making? Will they be useful for practical applications such as training experts to be creative in their specialty? In addition, there is of course the question as to what kind of AI is best suited for generating theories. We have provided the example of genetic programming, but many other techniques can be advanced as candidates, including adaptive production systems (Klahr et al., 1987) and deep learning (LeCun et al., 2015).

## Problems and Prospects

Recent developments in AI signal a new relationship between human and machine. Interesting albeit perhaps threatening questions are posed about our human nature and, specifically, the meaning of creativity. These include philosophical and ethical questions. Can a product be creative if it is conceived by a computer? If so, who owns the research? Should computer programs be listed as co-authors of scientific papers? How will the synergy between human and computer creativity evolve? Should some types of creativity – e.g., generating fake news for political aims – be curtailed or even banned?

These developments also raise significant questions about human rationality, as discussed above. In doing so, they highlight the magnificent achievements of some human creators, such as Wolfgang Amadeus Mozart or Pablo Picasso. In addition, they have substantial implications for creativity in science and the arts. Entirely new conceptual spaces might be explored, with computer programs either working independently or co-designing creative products with humans. In science – the focus of this perspective article – this might lead to the development of novel research strategies, methodologies, types of experiments, theories, and theoretical frameworks. Of particular interest is the possibility of mixing concepts and mechanisms between different subfields (e.g., between memory research and decision-making research), between different fields (e.g., psychology and chemistry), and even between science and the arts. As discussed above, there are also some new exciting opportunities for training. It is only with the aid of artificial creativity that we will break our mind-sets and reach a new understanding of human creativity.

## Author Contributions

Both authors conceptualized the paper. FG wrote the first draft of the paper and GS contributed to drafting its final version.

### Conflict of Interest Statement

The authors declare that the research was conducted in the absence of any commercial or financial relationships that could be construed as a potential conflict of interest.

## Footnotes

^1^It is notably difficult to define “creativity,” and a large number of definitions exist with little agreement among researchers (see e.g., Hennessey and Amabile, 2010). In this article, we focus on what Boden (1990) calls “historical creativity” (concerning products that are considered novel by society at large) rather than “psychological creativity” (concerning products that are novel only for the agent producing them). Thus, if Joe Bloggs for the first time of his life realizes that a brick can be used as a pen holder, this is psychological but not historical creativity. If he is the first ever to claim that a brick can be used as an abstract rendition of Beethoven’s 5th Symphony, this is both psychological and historical creativity according to Boden’s definition.

^2^While the aim of this *Perspective Article* is not to provide a review of the extensive literature on creativity in psychology and neuroscience, a few additional pointers might be helpful to the reader: Cristofori et al. (2018); Kaufman and Sternberg (2019); and Simonton (2014).
